# Supplementary material for: Single-dose bNAb cocktail or abbreviated ART post-exposure regimens achieve tight SHIV control without adaptive immunity
Source: Nat Commun. 2020 Jan 7;11:70. doi: 10.1038/s41467-019-13972-y (PMC6946664; doi:10.1038/s41467-019-13972-y)
Supplement: Supplementary file 1 — Supplementary Information [file 41467_2019_13972_MOESM1_ESM.pdf]

Single-dose bNAb cocktail or abbreviated ART post-exposure regimens achieve tight SHIV control without adaptive immunity

Shapiro et al.

Supplementary Figures

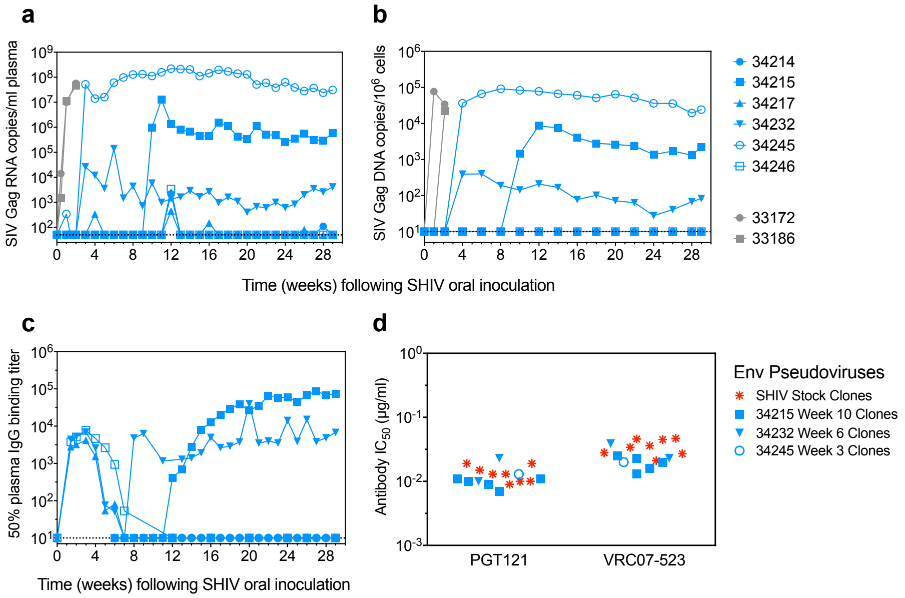

Supplementary Figure 1. Emergent virus is not resistant to bNAbs. One-month-old infant macaques (Group 2A, light blue symbols) were exposed orally with $SHIV_{SF162P3}$ and treated subcutaneously with 5 mg/kg PGT121 and 5 mg/kg VRC07-523 on days 2, 4, 7, and 10 after SHIV exposure. Contemporaneous age-matched control animals (gray symbols) were exposed with the same dose of SHIV and not treated with bNAbs. **a)** Plasma viral loads over time. **b)** Viral DNA in PBMC over time. Dotted lines indicate limits of detection**. c)** Plasma IgG binding titers against SF162 gp140 protein. Plasma dilution at which 50% of antigen is bound is shown. **d)** Neutralization of $SHIV_{SF162P3}$ stock and emergent clones from bNAb-treated animals. Env-pseudoviruses were cloned from the challenge SHIV stock (red symbols) or from plasma of three animals exposed to this SHIV stock and treated with 10 mg/kg bNAb cocktail on days 2, 4, 7, and 10 after exposure (light blue symbols). Time points were chosen based on sample availability and first detectable instance of plasma viremia in each animal. Env-pseudoviruses were tested for sensitivity to neutralization by the two bNAbs listed along the bottom. IC_{50}, concentration at 50% viral inhibition. Source data are provided as a Source Data file.

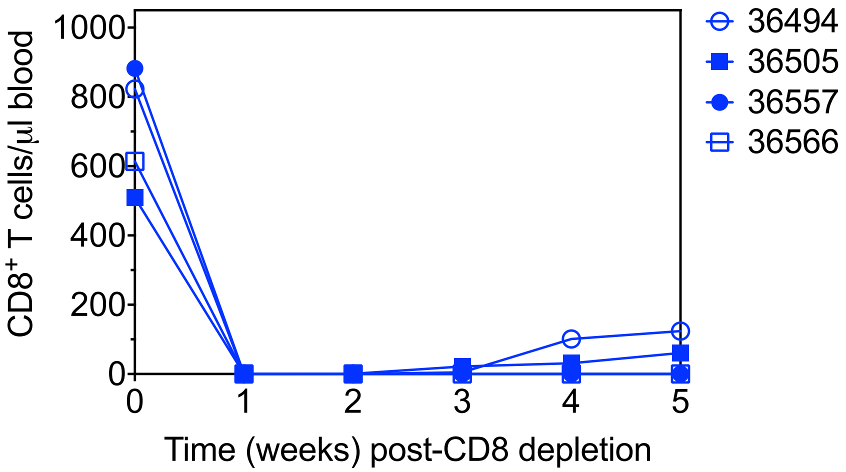

Supplementary Figure 2. CD8α depletion in four Group 2B animals (bNAbs at 48 hours). $CD8^+$ T cell counts in peripheral blood are shown for each animal. Time is measured in weeks after the first dose of depleting antibody. Group colors and individual animal symbols are consistent throughout the manuscript. Source data are provided as a Source Data file.

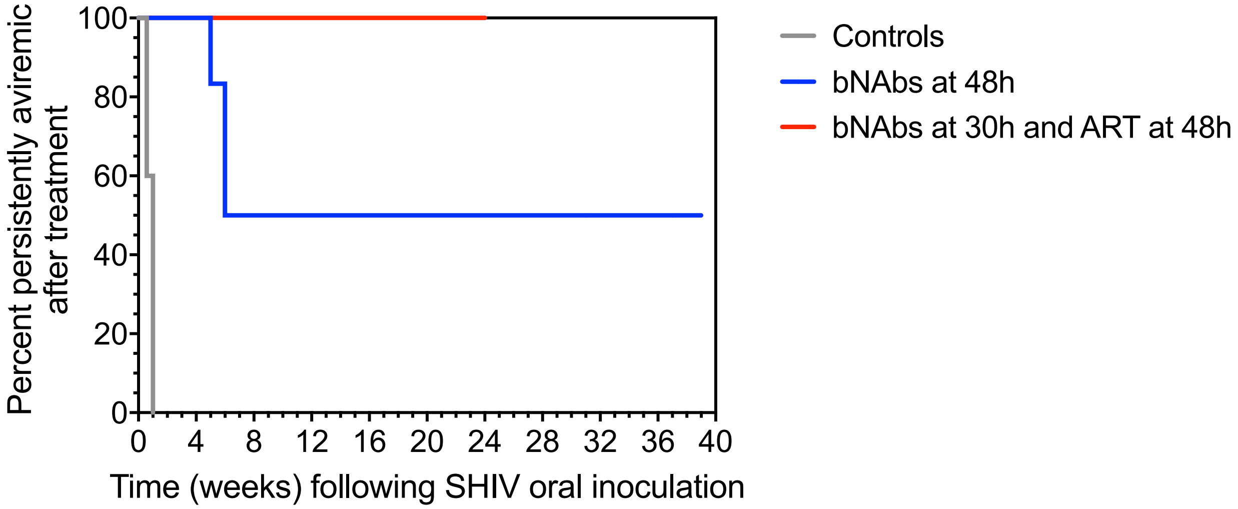

Supplementary Figure 3. Post-exposure bNAbs or ART prevent persistent viremia. Kaplan-Meier analysis of proportion of animals remaining persistently aviremic (defined as no more than one consecutive measurement of positive plasma viremia) after conclusion of treatment. Groups 3 (bNAbs at 30 hours, n = 6 animals) and 4 (ART at 48 hours, n = 6 animals) had a significantly smaller percentage of animals that became viremic than Group 1 (controls, n = 5 animals) (Log-rank test with Dunnett-Hsu correction for multiple comparisons, adjusted p = 0.0002 for both comparisons). Group 2B (bNAbs at 48 hours, n = 6 animals) was not significantly different from Group 1 (adjusted p = 0.0969). Source data are provided as a Source Data file.

a

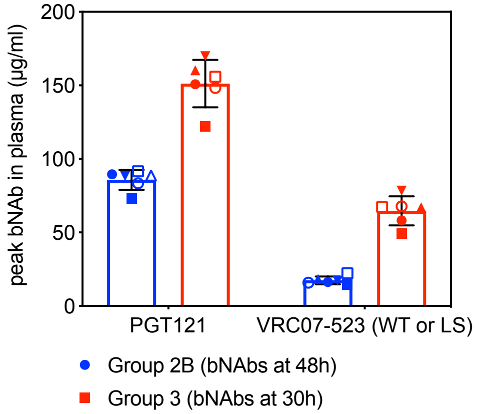

Source of Variation	% of total variation	P value	P value summary	Significant?	
Interaction	0.8632	0.0395	*	Yes	
bNAb	62.49	<0.0001	****	Yes	
Regimen	33.08	<0.0001	****	Yes	
ANOVA table	SS	DF	MS	F (DFn, DFd)	P value
Interaction	497.3	1	497.3	F (1, 20) = 4.851	P=0.0395
bNAb	36003	1	36003	F (1, 20) = 351.2	P<0.0001
Regimen	19060	1	19060	F (1, 20) = 185.9	P<0.0001
Residual	2051	20	102.5		

b

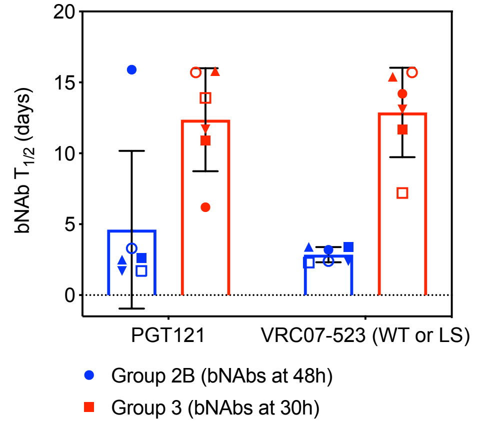

Source of Variation	% of total variation	P value	P value summary	Significant?	
Interaction	1.034	0.4568	ns	No	
bNAb	0.3099	0.6823	ns	No	
Regimen	62.73	<0.0001	****	Yes	
ANOVA table	SS	DF	MS	F (DFn, DFd)	P value
Interaction	7.820	1	7.820	F (1, 20) = 0.5757	P=0.4568
bNAb	2.344	1	2.344	F (1, 20) = 0.1725	P=0.6823
Regimen	474.4	1	474.4	F (1, 20) = 34.92	P<0.0001
Residual	271.7	20	13.58		

Supplementary Figure 4. Effects of bNAb and treatment regimen on concentration and half-life. The regimen factor includes 2 levels: the 4-dose regimen given starting at 48 hours and on days 4, 7, and 10 (Group 2B, n = 6 animals) and the single-dose regimen given at 30 hours (Group 3, n = 6 animals) after SHIV exposure. The bNAb factor includes 2 levels: PGT121 and VRC07-523 (wild-type [WT] for Group 2B, LS variant for Group 3). **a)** Peak concentration in plasma for each bNAb in each treatment group. **b)** bNAb half-life ($T_{1/2}$) in plasma for each bNAb in each treatment group. Blue symbols and bars, Group 2B. Red symbols and bars, Group 3. Individual animal values are plotted as symbols consistent with those used throughout the manuscript. The bar graph represents the mean. Error bars represent standard error of the mean (SEM). For peak concentration **(a)** and half-life **(b)**, results of the 2-way ANOVAs are shown in the tables at right. SS, sum of squares. DF, degrees of freedom. MS, mean squares. All statistical analyses are based on n = 6 animals per group. Source data are provided as a Source Data file.

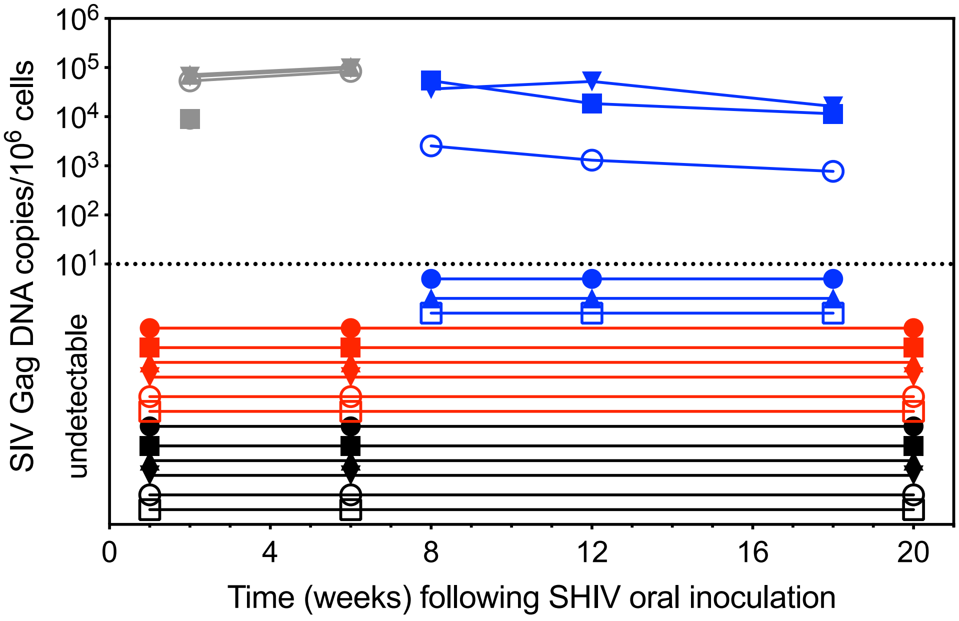

Supplementary Figure 5. Viral DNA in inguinal lymph nodes is stable over time. Gray, Group 1 (untreated controls). Blue, Group 2B (bNAbs at 48 hours). Red, Group 3 (bNAbs at 30 hours). Black, Group 4 (ART at 48 hours). Group colors and individual animal symbols are consistent throughout the manuscript. Total viral DNA levels were quantified in inguinal lymph nodes biopsied at the time points indicated. All samples shown below the dotted line at 10^1 DNA copies/10^6 cell equivalents were below the limit of detection. Source data are provided as a Source Data file.

Group	Animal ID	Spleen			Mixed Mesenteric Lymph Nodes		
		mean SIV gag DNA copies/million cells	IUPM (+TDF)	IUPM (-TDF)	mean SIV gag DNA copies/million cells	IUPM (+TDF)	IUPM (-TDF)
Group 1 (Controls)	36206	4408	*nd**	*nd**	1375	*nd**	*nd**
	36207	4053	*nd**	*nd**	16405	*nd**	*nd**
	37220	22021	*nd***	*nd***	71303	*nd***	*nd***
	37223	15148	375	718	105147	376	718
	37235	12307	9.0	29.0	73214	1668	2384
Group 2A (bNAbs at 48h)	34214	0.00	0.0	0.0	0.00	*nd*	*nd*
	34215	9760	4.7	718	7220	125	718
	34217	0.00	0.0	0.8	0.00	*nd*	*nd*
	34232	14	0.0	0.9	136	0.0	6.3
	34245	19159	5850	> 5851	54814	718	3521
	34246	0.13	0.0	0.0	0.00	*nd*	*nd*
Group 2B (bNAbs at 48h)	36494	0.00	0.0	0.0	0.00	4.0	0.0
	36505	1694	0.8	5850	2665	*nd***	*nd***
	36521	0.00	0.0	0.8	0.13	1.5	3.0
	36533	28432	718	1380	37780	2384	5850
	36557	1989	0.0	1.6	2724	0.0	2.7
	36566	0.30	0.0	0.0	0.47	0.0	0.0
Group 3 (bNAbs at 30h)	37331	0.00	0.0	0.0	0.00	0.0	0.0
	37341	0.00	0.0	0.0	0.00	0.0	0.8
	37342	0.00	0.0	0.0	0.00	0.0	0.0
	37359	0.00	0.0	0.0	0.00	0.0	0.8
	37363	0.00	0.0	0.0	0.00	0.0	0.0
	37364	0.00	0.0	0.0	0.00	0.0	0.0
Group 4 (ART at 48h)	37389	0.00	0.0	0.0	0.00	0.0	0.0
	37390	0.00	0.0	0.0	0.00	0.0	0.0
	37427	0.00	0.0	0.0	0.00	0.0	0.0
	37480	0.00	0.0	1.5	0.00	0.0	0.0
	37481	0.00	0.0	3.1	0.00	0.0	4.5
	37482	0.00	0.0	0.0	0.00	0.8	6.0

Supplementary Figure 6. Inducible replication-competent virus in spleen and lymph nodes. TDF, tenofovir disoproxil fumarate. IUPM, infectious units per million cells. Spleen and mixed mesenteric lymph nodes were collected at necropsy and replication-competent virus was measured in CD4-enriched single cell suspensions using a TZM-bl-based viral outgrowth (TZA) assay. Cells were stimulated *in vitro* either in the presence of TDF (+TDF) to limit *de novo* replication during cell stimulation, or in its absence (-TDF) to increase assay sensitivity. Values for the -TDF condition are an overestimate and not truly quantitative, and are only reported to give an idea of positivity relative to the +TDF condition. *nd*, not done. One asterisk (*), tissue sample not collected. Two asterisks (**), assay not possible due to microbial contamination. Source data are provided as a Source Data file.

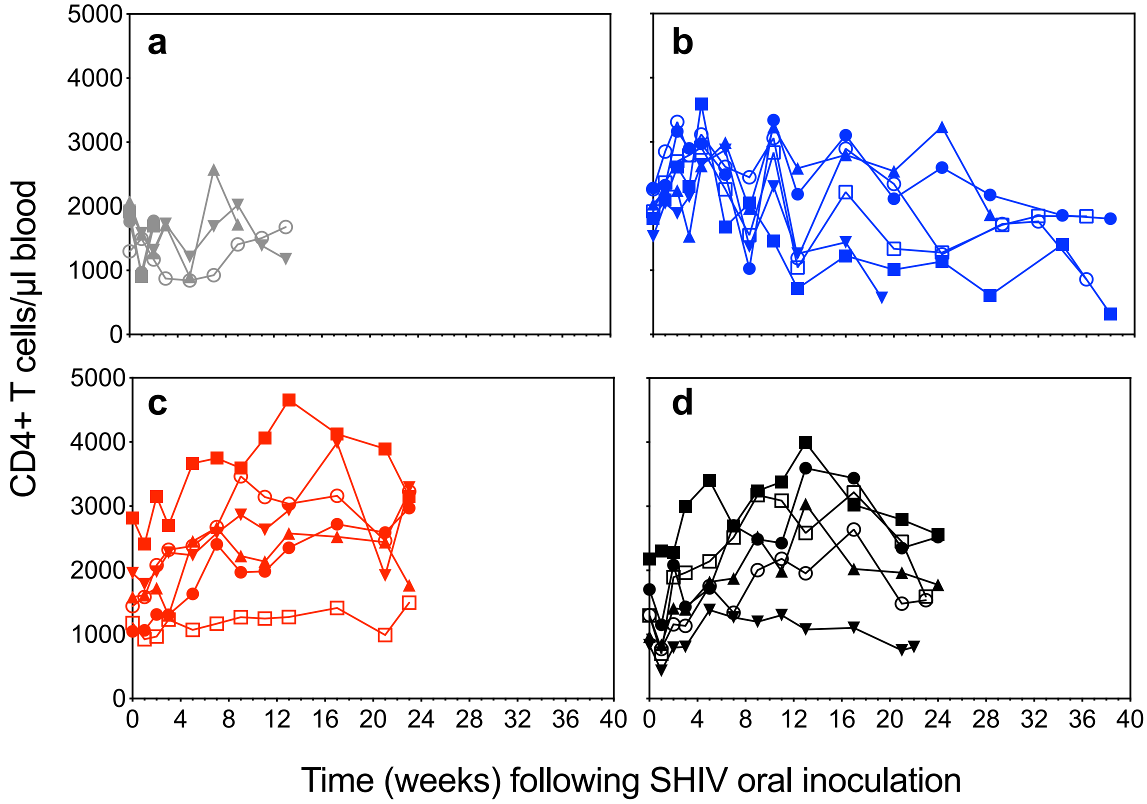

Supplementary Figure 7. Longitudinal CD4^{+} T cell counts. a) Group 1 (untreated controls). **b)** Group 2B (bNAbs at 48 hours). **c)** Group 3 (bNAbs at 30 hours). **d)** Group 4 (ART at 48 hours). Group colors and individual animal symbols are consistent throughout the manuscript. Source data are provided as a Source Data file.

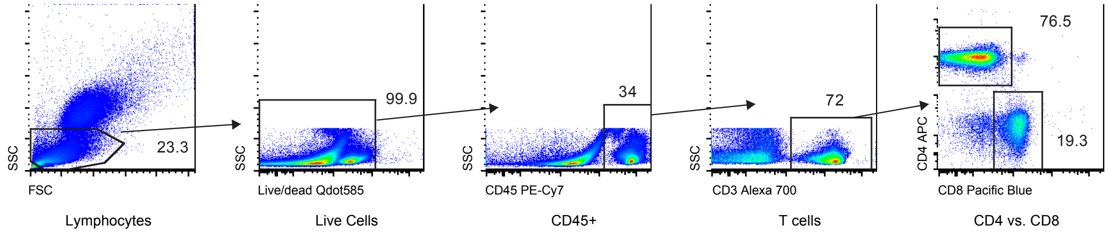

Supplementary Figure 8. Gating strategy for CD4^{+} and CD8^{+} T cell counts by flow cytometry. After gating on lymphocytes, singlets, and live cells, CD4^{+} T cells were defined as CD45^{+} CD3^{+} CD4^{+} CD8^{-}, and CD8^{+} T cells were defined as CD45^{+} CD3^{+} CD4^{-} CD8^{+}. Gating for a representative peripheral blood sample is shown.

Supplementary Tables

Supplementary Table 1. Titration of $SHIV_{SF162P3}$ stocks *in vivo* by single dose oral challenge in infant rhesus macaques.

Study Groups	SHIV Virus Stock	Challenge Dose (ml virus)	Challenge Dose ($TCID_{50}$ measured in rhesus PBMC)	Of untreated control animals, number viremic/total (%)	Of untreated control animals, number with sustained high viremia/total (%)
2A	$SHIV_{SF162P3}$ (NIH)	0.5	885	2/2 (100%)	2/2 (100%)
1, 2B, 3, 4	$SHIV_{SF162P3}$ (OHSU-2017)	2	40960	5/5 (100%)	3/5 (60%)
x	$SHIV_{SF162P3}$ (OHSU-2017)	1	20480	2/2 (100%)	1/2 (50%)
x	$SHIV_{SF162P3}$ (OHSU-2017)	0.5	10240	4/6 (67%)	3/6 (50%)
x	$SHIV_{SF162P3}$ (OHSU-2017)	0.2	4096	0/2 (0%)	0/2 (0%)
x	$SHIV_{SF162P3}$ (OHSU-2017)	0.1	2048	1/4 (25%)	1/4 (25%)

x = this viral dose was not used in this study; it was only performed as part of an *in vivo* titration experiment to establish the appropriate viral dose to infect infant macaques for treatment studies including this one.

Supplementary Table 2. Viral DNA in tissues at time of death in Group 1 (untreated controls).

Animal ID (sex)	36206 (F)	36207 (M)	37220 (F)	37223 (M)	37235 (M)
Buccal Mucosa	262.54	63.17	x	x	x
Pharyngeal Mucosa	1108.26	1255.10	x	x	x
Esophagus	82.24	117.17	x	x	x
Stomach	604.22	2398.24	x	x	x
Duodenum	356.97	1569.49	12431.92	4920.54	4440.85
Jejunum	1011.80	1271.29	4499.76	2181.51	4676.33
Ileum	1947.42	1999.09	6788.91	3806.57	10624.47
Cecum	1081.90	1236.36	4916.87	3984.19	2029.65
Colon Ascending	1657.01	1537.68	x	x	x
Colon Transverse	1212.37	1427.19	x	x	x
Colon Descending	2394.72	1613.70	4208.47	25045.60	3424.81
Rectum	1571.66	634.25	6223.62	4642.02	2569.94
Tonsil	5760.80	4985.08	17060.22	5248.68	4952.87
Submandibular LN	6534.83	9782.95	51142.63	23191.66	77175.42
Retropharyngeal LN	658.26	6367.97	86092.38	55822.68	98429.89
Tracheobronchial LN	8868.62	11465.06	76229.20	39043.67	35993.26
Axillary LN	6008.74	10683.62	49678.89	25303.49	42952.55
Mixed Mesenteric LN	1374.58	16405.11	71303.26	105147.23	73214.19
Iliosacral LN	6136.82	6889.34	25386.19	35225.85	38236.22
Inguinal LN	8736.87	8999.33	49972.94	48075.27	47787.75
Spleen	4407.60	4053.21	22021.45	15148.00	12306.68
Mixed Repro Tract	107.86	47.51	x	x	x
Lungs	222.55	226.98	x	x	x
Thymus	83.10	38.59	x	x	x
Adrenal Gland	20.80	32.26	x	x	x
Pancreas	6389.00	6847.42	x	x	x
Kidney	33.69	24.57	x	x	x
Liver	x	x	x	x	x
Bladder	193.19	245.75	x	x	x
Thyroid	22.54	50.51	x	x	x
Cerebellum Brain	0.00	0.00	x	x	x
Cerebrospinal Fluid*	338.54	727.46	6530.54	41145.22	1716.50

Results are mean SIV Gag DNA copies per 10^6 total cells, except where indicated by a * symbol
x = not collected
* units are RNA copies/ml

Supplementary Table 3. Viral DNA in tissues at time of death in Group 2A (bNAbs at 48 hours).

Animal ID (sex)	34214 (F)	34215 (M)	34217 (F)	34232 (M)	34245 (M)	34246 (M)
Buccal Mucosa	0.00	7.97	0.00	0.13	34.45	0.00
Pharyngeal Mucosa	x	878.96	x	1.11	8695.22	x
Esophagus	0.00	1034.02	0.00	10.96	538.82	0.68
Stomach	0.00	12.27	0.13	7.67	961.13	0.00
Duodenum	0.00	493.03	0.00	9.99	7162.96	0.00
Jejunum	0.00	736.49	1.21	71.57	55182.56	1.21
Ileum	0.00	116.73	0.13	165.05	7605.49	0.00
Cecum	0.00	718.02	0.00	98.37	9979.20	0.00
Colon Ascending	0.00	3072.88	0.00	77.70	32202.49	0.00
Colon Transverse	0.00	4478.30	0.00	35.02	27767.40	0.13
Colon Descending	0.00	1866.49	0.00	26.77	29237.91	0.30
Rectum	0.00	2186.43	2.26	53.71	24165.32	0.00
Tonsil	0.00	22497.36	0.00	200.81	491.05	0.13
Submandibular LN	0.00	11322.05	0.00	514.18	59377.54	0.00
Retropharyngeal LN	0.00	13084.81	0.00	401.45	36491.34	0.00
Tracheobronchial LN	0.00	6864.54	0.00	139.44	79775.15	0.47
Axillary LN	0.00	9239.09	0.00	118.07	53829.92	32.56
Mixed Mesenteric LN	0.00	7220.38	0.00	135.70	54814.27	0.00
Iliosacral LN	0.00	9639.79	0.00	644.49	71868.11	0.13
Inguinal LN	0.00	12049.44	0.00	458.11	41839.97	0.00
Spleen	0.00	9759.76	0.00	13.76	19158.69	0.13
Mixed Repro Tract	0.00	194.51	0.00	0.00	254.45	0.13
Lungs	0.00	0.25	0.00	0.19	445452.25	0.13
Thymus	0.00	33.04	0.00	0.00	38110.55	0.00
Adrenal Gland	0.00	17.20	0.00	0.00	5040.59	0.00
Pancreas	0.00	4.41	0.00	0.00	3263.52	0.00
Kidney	0.00	18.68	0.00	0.00	592.71	0.00
Liver	0.00	15.51	0.00	0.00	5706.21	0.00
Bladder	0.00	31.64	0.00	2.08	361.87	0.00
Thyroid	0.00	24.41	x	0.00	1389.77	0.13
Cerebellum Brain	0.00	2.70	0.00	0.00	40.25	0.00
Cerebrospinal Fluid*	0.00	2805.95	0.00	153.34	1460.37	0.00

Results are mean SIV Gag DNA copies per 10^6 total cells, except where indicated by a * symbol

x = not collected

* units are RNA copies/ml

Supplementary Table 4. Viral DNA in tissues at time of death in Group 2B (bNAbs at 48 hours).

Animal ID (sex)	36494 (F)	36505 (F)	36521 (F)	36533 (F)	36557 (F)	36566 (F)
Buccal Mucosa	x	x	x	x	x	x
Pharyngeal Mucosa	x	x	x	x	x	x
Esophagus	x	x	x	x	x	x
Stomach	x	x	x	x	x	x
Duodenum	0.13	130.40	12.39	1129.32	150.63	0.91
Jejunum	0.00	120.62	0.00	1667.05	193.70	0.00
Ileum	0.00	1241.93	0.00	1140.22	4093.82	0.00
Cecum	0.00	371.14	0.00	1825.68	7591.03	0.13
Colon Ascending	x	x	x	x	x	x
Colon Transverse	x	x	x	x	x	x
Colon Descending	0.00	360.16	0.68	1468.65	2846.26	0.47
Rectum	0.00	524.89	0.00	5379.52	2478.46	0.00
Tonsil	0.00	3105.62	0.00	3486.99	3572.91	0.00
Submandibular LN	0.00	4831.04	0.00	51478.13	1905.10	0.00
Retropharyngeal LN	0.00	3133.38	0.00	23599.91	1700.90	0.00
Tracheobronchial LN	1.25	4456.31	0.00	3833.70	2377.28	0.00
Axillary LN	0.00	3431.00	0.00	50070.11	2973.55	0.00
Mixed Mesenteric LN	0.00	2665.00	0.13	37780.08	2723.72	0.47
Iliosacral LN	0.00	3154.65	0.00	29909.66	2591.30	0.00
Inguinal LN	0.00	3136.97	0.00	28897.06	3002.55	0.47
Spleen	0.00	1694.08	0.00	28431.70	1988.60	0.30
Mixed Repro Tract	x	x	x	x	x	x
Lungs	x	x	x	x	x	x
Thymus	x	x	x	x	x	x
Adrenal Gland	x	x	x	x	x	x
Pancreas	x	x	x	x	x	x
Kidney	x	x	x	x	x	x
Liver	x	x	x	x	x	x
Bladder	x	x	x	x	x	x
Thyroid	x	x	x	x	x	x
Cerebellum Brain	x	x	x	x	x	x
Cerebrospinal Fluid*	0.00	4315.11	0.00	1283.41	13830.52	0.00

Results are mean SIV Gag DNA copies per 10^6 total cells, except where indicated by a * symbol

x = not collected

* units are RNA copies/ml

Supplementary Table 5. Viral DNA in tissues at time of death in Group 3 (bNAbs at 30 hours).

Animal ID (sex)	37331 (M)	37341 (F)	37342 (F)	37359 (M)	37363 (M)	37364 (F)
Buccal Mucosa	x	x	x	x	x	x
Pharyngeal Mucosa	x	x	x	x	x	x
Esophagus	x	x	x	x	x	x
Stomach	x	x	x	x	x	x
Duodenum	0.00	0.00	0.00	0.00	0.00	0.00
Jejunum	0.00	0.00	0.00	0.00	0.00	0.00
Ileum	0.00	0.00	0.00	0.00	0.00	0.00
Cecum	0.00	0.00	0.00	0.00	0.00	0.00
Colon Ascending	x	x	x	x	x	x
Colon Transverse	x	x	x	x	x	x
Colon Descending	0.00	0.00	0.00	0.00	0.00	0.00
Rectum	0.00	0.00	0.00	0.00	0.00	0.00
Tonsil	0.00	0.00	0.00	0.00	0.00	0.00
Submandibular LN	0.00	0.00	0.00	0.00	0.00	0.00
Retropharyngeal LN	0.00	0.00	0.00	0.00	0.00	0.00
Tracheobronchial LN	0.00	0.00	0.00	0.00	0.00	0.00
Axillary LN	0.00	0.00	0.00	0.00	0.00	0.00
Mixed Mesenteric LN	0.00	0.00	0.00	0.00	0.00	0.00
Iliosacral LN	0.00	0.00	0.00	0.00	0.00	0.00
Inguinal LN	0.00	0.00	0.00	0.00	0.00	0.00
Spleen	0.00	0.00	0.00	0.00	0.00	0.00
Mixed Repro Tract	x	x	x	x	x	x
Lungs	x	x	x	x	x	x
Thymus	x	x	x	x	x	x
Adrenal Gland	x	x	x	x	x	x
Pancreas	x	x	x	x	x	x
Kidney	x	x	x	x	x	x
Liver	x	x	x	x	x	x
Bladder	x	x	x	x	x	x
Thyroid	x	x	x	x	x	x
Cerebellum Brain	x	x	x	x	x	x
Cerebrospinal Fluid*	0.00	0.00	0.00	0.00	0.00	0.00

Results are mean SIV Gag DNA copies per 10^6 total cells, except where indicated by a * symbol

x = not collected

* units are RNA copies/ml

Supplementary Table 6. Viral DNA in tissues at time of death in Group 4 (ART at 48 hours).

Animal ID (sex)	37389 (F)	37390 (M)	37427 (F)	37480 (F)	37481 (M)	37482 (M)
Buccal Mucosa	x	x	x	x	x	x
Pharyngeal Mucosa	x	x	x	x	x	x
Esophagus	x	x	x	x	x	x
Stomach	x	x	x	x	x	x
Duodenum	0.00	0.00	0.00	0.00	0.00	0.00
Jejunum	0.00	0.00	0.00	0.00	0.00	0.00
Ileum	0.00	0.00	0.00	0.00	0.00	0.00
Cecum	0.00	0.00	0.00	0.00	0.00	0.00
Colon Ascending	x	x	x	x	x	x
Colon Transverse	x	x	x	x	x	x
Colon Descending	0.00	0.00	0.00	0.00	0.00	0.00
Rectum	0.00	0.00	0.00	0.00	0.00	0.00
Tonsil	0.00	0.00	0.00	0.00	0.00	0.00
Submandibular LN	0.00	0.00	0.00	0.00	0.00	0.00
Retropharyngeal LN	0.00	0.00	0.00	0.00	0.00	0.00
Tracheobronchial LN	0.00	0.13	0.00	0.00	0.00	0.00
Axillary LN	0.00	0.00	0.00	0.00	0.00	0.00
Mixed Mesenteric LN	0.00	0.00	0.00	0.00	0.00	0.00
Iliosacral LN	0.13	0.00	0.00	0.00	0.00	0.00
Inguinal LN	0.00	0.00	0.00	0.00	0.00	0.00
Spleen	0.00	0.00	0.00	0.00	0.00	0.00
Mixed Repro Tract	x	x	x	x	x	x
Lungs	x	x	x	x	x	x
Thymus	x	x	x	x	x	x
Adrenal Gland	x	x	x	x	x	x
Pancreas	x	x	x	x	x	x
Kidney	x	x	x	x	x	x
Liver	x	x	x	x	x	x
Bladder	x	x	x	x	x	x
Thyroid	x	x	x	x	x	x
Cerebellum Brain	x	x	x	x	x	x
Cerebrospinal Fluid*	0.00	0.00	0.00	0.00	0.00	0.00

Results are mean SIV Gag DNA copies per 10^6 total cells, except where indicated by a * symbol

x = not collected

* units are RNA copies/ml

Supplementary Table 7. Spearman correlations between tissue viral DNA copies and plasma viremia for Groups 1, 2B, 3, and 4.

Tissue	PVL peak Spearman Rho	PVL peak p-value	PVL final Spearman Rho	PVL final p-value	PVL AUC Spearman Rho*	PVL AUC p-value*
Tonsil	0.83	<.0001	0.96	<.0001	0.82	<.0001
Submandibular LN	0.85	<.0001	0.99	<.0001	0.82	<.0001
Retropharyngeal LN	0.85	<.0001	0.89	<.0001	0.82	<.0001
Tracheobronchial LN	0.79	<.0001	0.99	<.0001	0.68	0.0007
Axillary LN	0.86	<.0001	0.92	<.0001	0.83	<.0001
Mixed Mesenteric LN	0.78	<.0001	1	<.0001	0.76	<.0001
Iliosacral LN	0.87	<.0001	0.92	<.0001	0.86	<.0001
Inguinal LN	0.85	<.0001	0.95	<.0001	0.83	<.0001
Spleen	0.85	<.0001	0.96	<.0001	0.83	<.0001
Lymphoid tissues average**	0.81	<.0001	0.88	<.0001	0.72	0.0002
Duodenum	0.79	<.0001	0.91	<.0001	0.71	0.0003
Jejunum	0.85	<.0001	0.99	<.0001	0.82	<.0001
Ileum	0.82	<.0001	0.95	<.0001	0.8	<.0001
Cecum	0.81	<.0001	0.92	<.0001	0.81	<.0001
Colon Descending	0.75	<.0001	0.9	<.0001	0.75	<.0001
Rectum	0.84	<.0001	0.99	<.0001	0.83	<.0001
GI tract tissues average***	0.77	<.0001	0.89	<.0001	0.7	0.0004

PVL, plasma viral load (RNA copies/ml)
PVL peak, maximum plasma viral load (RNA copies/ml) for each animal.
PVL AUC, area under the curve for plasma viral load.
LN, lymph node.
GI tract, gastrointestinal tract.
Except for PVL AUC (see below), data from a total of n = 23 animals were included in the correlation analyses.
* For PVL AUC analysis, data after 10 weeks was censored for all animals, and animals 36206 and 36207 were excluded because they were sacrificed prior to 10 weeks. A total of 21 animals were included in the PVL AUC analysis.
** Lymphoid tissues include tonsil, all lymph nodes listed in the table, and spleen.
*** GI tract tissues include duodenum, jejunum, ileum, cecum, colon descending, and rectum.

Supplementary Table 8. Clinical histories and pathologic findings for Group 1 (untreated controls).

Animal ID (Sex)	Age at termination (days)	Opportunistic infections, pathologic diagnoses, and clinical history
36206 (F)	38	Gross and microscopic findings normal for age. Unremarkable clinical history.
36207 (M)	36	Mild pharyngitis and interstitial pneumonia consistent with mild viral respiratory infection. Clinical history of periocular erythema, ventral abdominal rash, single episode of diarrhea.
37220 (F)	100	Minimal pancreatic duct cryptosporidiosis, mild membranous glomerulopathy, mild portal hepatitis. Chronic-active typhlocolitis. Clinical episode of diarrhea associated with *Campylobacter coli*.
37223 (M)	128	Multiple opportunistic infections and SHIV-related pathology including SHIV giant cell pneumonia; acute bronchopneumonia; cytomegaloviral interstitial pneumonia, orchitis, and sialoadenitis; proliferative choledochitis/cholecystitis attributed to *Enterocytozoon bieneusi*; mild membranous glomerulopathy. Chronic-active typhlocolitis. Thymic depletion. Clinical history of diarrhea, abdominal bloating, cough, and facial rash. Early termination due to clinical pneumonia.
37235 (M)	129	Several opportunistic infections including enteric and pancreatic duct cryptosporidiosis; proliferative choledochitis attributed to *Enterocytozoon bieneusi*, mild membranous glomerulopathy. Mild chronic-active typhlocolitis. Unremarkable clinical history.

Supplementary Table 9. Clinical histories and pathologic findings for Group 2A (bNAbs at 48 hours).

Animal ID (Sex)	Age at termination (days)	Opportunistic infections, pathologic diagnoses and clinical history
34214 (F)	250	Gross and microscopic findings normal for age. Unremarkable clinical history.
34215 (M)	246	Mild eosinophilic and granulomatous mediastinitis, thymitis. Unremarkable clinical history.
34217 (F)	246	Gross and microscopic findings normal for age. Unremarkable clinical history.
34232 (M)	236	Gross and microscopic findings normal for age. Unremarkable clinical history.
34245 (M)	237	Multiple opportunistic infections and SHIV-related pathology including flagellate gastritis; proliferative choledochitis and cholecystitis due to *Enterocytozoon bieneusi*; Pneumocystis pneumonia; adenoviral enteritis; intracytoplasmic agyrophilic bacteria in stomach and duodenum; enteropathy with attaching and effacing *Escherichia coli*; proliferative proctitis with flagellated protozoa; necrotizing granulomatous lymphadenitis due to *Spironucleus* sp.; mild membranous glomerulopathy; and SHIV giant cells in lymph nodes and lung. Chronic-active proliferative typhlocolitis. Widespread lymphoid depletion. Clinical history of erythematous rash; delayed growth and weight gain (35% below norm).
34246 (M)	240	Dermatitis with Malassezia sp yeast. Unremarkable clinical history.

Supplementary Table 10. Clinical histories and pathologic findings for Group 2B (bNAbs at 48 hours).

Animal ID (Sex)	Age at termination (days)	Opportunistic infections, pathologic diagnoses and clinical history
36494 (F)	305	Gross and microscopic findings normal for age. Unremarkable clinical history.
36505 (F)	303	Focal lymphocytic perivascular infiltrate in brainstem and cellulitis of chin. Moderate chronic-active typhlocolitis. Clinical history of intermittent diarrhea associated with *Campylobacter coli* and delayed growth.
36521 (F)	243	Gross and microscopic findings normal for age. Unremarkable clinical history.
36533 (F)	173	Mild membranous glomerulopathy. Moderate chronic-active typhlocolitis. Clinical history of delayed growth, maculopapular rash in inguinal region and ventral abdomen.
36557 (F)	293	Mild inflammation in meninges, kidney, liver, and large intestine. Unremarkable clinical history.
36566 (F)	289	Mild chronic-active typhlocolitis. Clinical history of diarrhea with positive fecal culture for Campylobacter coli and mildly delayed growth rate

Supplementary Table 11. Clinical histories and pathologic findings for Group 3 (bNAbs at 30 hours).

Animal ID (Sex)	Age at termination (days)	Opportunistic infections, pathologic diagnoses and clinical history
37331 (M)	187	Mild segmental chronic-active typhlocolitis. Unremarkable clinical history.
37341 (F)	183	Gross and microscopic findings normal for age. Unremarkable clinical history.
37342 (F)	183	Mild inflammation in lung, liver, and large intestine. Clinical history of intermittently delayed growth.
37359 (M)	194	Moderate chronic-active typhlocolitis. Clinical history of intermittent diarrhea, emesis and delayed growth rate.
37363 (M)	196	Mild multifocal granulomatous pneumonia. Unremarkable clinical history.
37364 (F)	193	Gross and microscopic findings normal for age. Unremarkable clinical history.

Supplementary Table 12. Clinical histories and pathologic findings for Group 4 (ART at 48 hours).

Animal ID (Sex)	Age at termination (days)	Opportunistic infections, pathologic diagnoses and clinical history
37389 (F)	199	Mild chronic-active typhlocolitis. Single episode of diarrhea.
37390 (M)	198	Gross and microscopic findings normal for age. Unremarkable clinical history.
37427 (F)	195	Gross and microscopic findings normal for age. Unremarkable clinical history.
37480 (F)	184	Mild chronic-active typhlocolitis. Clinical episode of diarrhea associated with *Campylobacter jejuni*.
37481 (M)	195	Gross and microscopic findings normal for age. Unremarkable clinical history.
37482 (M)	198	Gross and microscopic findings normal for age. Clinical history of mild papular rash.

Supplementary Table 13. List of primers used in this study.

**Primer Name**	**Primer Sequence (5' – 3')**	**Purpose**
SGAG21 forward	GTCTGCGTCATPTGGTGCATTC	Viral load quantitation in plasma, PBMC, tissues
SGAG22 reverse	CACTAGKTGTCTCTGCACTATPTGTTTTG	Viral load quantitation in plasma, PBMC, tissues
pSGAG23	(FAM)-CTTCPTCAGTKTGTTTCACTTTCTCTTCTGCG-(BHQ1)	Viral load quantitation in plasma, PBMC, tissues
SIVnestF01	GATTTGGATTAGCAGAAAGCCTGTTG	Viral load quantitation in tissues (pre-amplification)
SIVnestR01	GTTGGTCTACTTGTTTTTGGCATAGTTTC	Viral load quantitation in tissues (pre-amplification)
BGenv3out	GGCCTCACTGATACCCCTACC	Single genome analysis (cDNA synthesis, first round of PCR)
BGenv5out	GCTATACCGCCCTCTAGAAGC	Single genome analysis (first round of PCR)
P3envB5in_NheI	GATCGCTAGCGTATGGGTCACAGTCT	Single genome analysis (second round of PCR)
P3envB3in_MLuI	GATCGACGCGTATCCATATTGTAGGT	Single genome analysis (second round of PCR)
SK1	GATCCTTAAGGCAGCGGCAGAAGAA	Single genome analysis (colony PCR)
SK6	GATCGTGTATGGCTGATTATGATGAT	Single genome analysis (colony PCR, gp160 sequencing)
218	ATCATTACACTTTAGAATCGC	Single genome analysis (gp160 sequencing)
ED5P3mod	ATGGGATCAAAGTCTAGAGCCATGTG	Single genome analysis (gp160 sequencing)
KK1	GCACAGTACAATGTACACATGGAA	Single genome analysis (gp160 sequencing)
env8R	CACAATCCTCGCTGCAATCAAG	Single genome analysis (gp160 sequencing)
env6For	GAATTGGATAAGTGGGCAAG	Single genome analysis (gp160 sequencing)
SK5	GATCGCCGTGAATTTAAGGGACGCTG	Single genome analysis (gp160 sequencing)
HTTS2-FW	GCCTYATYGCGTTAACGAGC	Evaluation of pathology – detection of *Spironucleus* sp. (first round of PCR)
HTTS2-RV	GACGGGCGGTGTRTACAAAR	Evaluation of pathology – detection of *Spironucleus* sp. (first round of PCR)
Ssp3-FW	AGACGGCCAGCCCCCCGGGC	Evaluation of pathology – detection of *Spironucleus* sp. (second round of PCR)
Ssp3-RV	GGTGTGGCCGCGGCCGGAGC	Evaluation of pathology – detection of *Spironucleus* sp. (second round of PCR)
