# Supplementary material for: Single-dose bNAb cocktail or abbreviated ART post-exposure regimens achieve tight SHIV control without adaptive immunity
Source: Nat Commun. 2020 Jan 7;11:70. doi: 10.1038/s41467-019-13972-y (PMC6946664; doi:10.1038/s41467-019-13972-y)
Supplement: Supplementary file 3 — Reporting Summary [file 41467_2019_13972_MOESM3_ESM.pdf]

nature research

Corresponding author(s): Nancy L. Haigwood, Ph.D.

Last updated by author(s): Nov 25, 2019

Reporting Summary

Nature Research wishes to improve the reproducibility of the work that we publish. This form provides structure for consistency and transparency in reporting. For further information on Nature Research policies, see Authors & Referees and the Editorial Policy Checklist .

Statistics

For all statistical analyses, confirm that the following items are present in the figure legend, table legend, main text, or Methods section.

n/a	Confirmed	
☐	☒	The exact sample size (*n*) for each experimental group/condition, given as a discrete number and unit of measurement
☐	☒	A statement on whether measurements were taken from distinct samples or whether the same sample was measured repeatedly
☐	☒	The statistical test(s) used AND whether they are one- or two-sided<br>*Only common tests should be described solely by name; describe more complex techniques in the Methods section.*
☒	☐	A description of all covariates tested
☐	☒	A description of any assumptions or corrections, such as tests of normality and adjustment for multiple comparisons
☐	☒	A full description of the statistical parameters including central tendency (e.g. means) or other basic estimates (e.g. regression coefficient) AND variation (e.g. standard deviation) or associated estimates of uncertainty (e.g. confidence intervals)
☐	☒	For null hypothesis testing, the test statistic (e.g. *F*, *t*, *r*) with confidence intervals, effect sizes, degrees of freedom and *P* value noted<br>*Give P values as exact values whenever suitable.*
☒	☐	For Bayesian analysis, information on the choice of priors and Markov chain Monte Carlo settings
☒	☐	For hierarchical and complex designs, identification of the appropriate level for tests and full reporting of outcomes
☐	☒	Estimates of effect sizes (e.g. Cohen's *d*, Pearson's *r*), indicating how they were calculated

Our web collection on statistics for biologists contains articles on many of the points above.

Software and code

Policy information about availability of computer code

Data collection	SoftMax Pro version 5.4 (Molecular Devices LLC, San Jose, CA, USA) for reading ELISA microplates; Becton Dickenson LSR II cytometer with BD FACSDIVA version 8.0.1 software (BD Biosciences, San Jose, CA, USA) for flow cytometry; AID EliSpot Reader version 6 software (Autoimmun Diagnostika GmbH, Strassberg, Germany) for reading ELISPOT plates
Data analysis	Excel v15.24 (Microsoft Inc., Redmond, WA, USA); Prism 8 (GraphPad Software Inc., San Diego, CA, USA); FlowJo v9.9.5 (Tree Star Inc., Ashland, OR, USA); SAS v9.4 (SAS Institute Inc., Cary, NC, USA)

For manuscripts utilizing custom algorithms or software that are central to the research but not yet described in published literature, software must be made available to editors/reviewers. We strongly encourage code deposition in a community repository (e.g. GitHub). See the Nature Research guidelines for submitting code & software for further information.

Data

Policy information about availability of data

All manuscripts must include a data availability statement. This statement should provide the following information, where applicable:

- Accession codes, unique identifiers, or web links for publicly available datasets
- A list of figures that have associated raw data
- A description of any restrictions on data availability

The data shown in the plots in Figure 4, along with data for additional tissues not shown in Figure 4, is provided in Supplementary Tables 2-6. Raw data underlying Figures 2, 3, and 5 and Supplementary Figures 1-7 are provided as Source Data files. All other data are available from the corresponding author on reasonable request.

October 2018

Field-specific reporting

Please select the one below that is the best fit for your research. If you are not sure, read the appropriate sections before making your selection.

- [x] Life sciences
- [ ] Behavioural & social sciences
- [ ] Ecological, evolutionary & environmental sciences

For a reference copy of the document with all sections, see nature.com/documents/nr-reporting-summary-flat.pdf

Life sciences study design

All studies must disclose on these points even when the disclosure is negative.

Sample size	Sample sizes were calculated assuming a 2-sided hypothesis test, with 80% power and alpha=0.05. The number of macaques needed in each experimental group to be able to detect significant differences in PVL have been determined. Experimental parameters were modeled using the virus stock SHIVSF162P3 in M. mulatta newborns, n=6. Week 2 data are used for the peak PVL in the controls (mean=7.650 log10 and SD=0.486 log10), and week 12 PVL for the set point (mean=6.456 log10 and SD=1.631 log10). Sample size needed in each group to see a halving of the set point viral load (i.e., a drop on average to 3.26 log10) is six.
Data exclusions	Flow cytometry data on peripheral blood B cell counts and T cell memory subsets were collected, but were excluded from the manuscript because they were not relevant to the main focus of the study. Virus neutralization was measured for plasma samples, but the data were redundant with the binding results measured by ELISA (Fig. 5) and thus were excluded. These data are available from the corresponding author on reasonable request.
Replication	Replicated data are noted in the description for each assay in Methods. Each assay was run with a validated standard.
Randomization	Macaques were assigned to studies as they were born and placed into the nursery for hand-rearing, and thus they were assigned randomly with respect to sex or body weight.
Blinding	Animal care technicians, veterinarians, and investigators assessing and documenting pathology were blinded to the animals' group assignments. For quantification of viral loads in blood, cells and tissues, samples were identified by type, animal number and date of collection and provided in bulk without keys to group assignments. No blinding was done for assays measuring humoral immune responses and viral outgrowth. For T cell responses, the experimenters had no access to the key for animal number as a sample identifier, nor any information on infection status.

Reporting for specific materials, systems and methods

We require information from authors about some types of materials, experimental systems and methods used in many studies. Here, indicate whether each material, system or method listed is relevant to your study. If you are not sure if a list item applies to your research, read the appropriate section before selecting a response.

Materials & experimental systems

n/a	Involved in the study	
[ ]	[x]	Antibodies
[ ]	[x]	Eukaryotic cell lines
[x]	[ ]	Palaeontology
[ ]	[x]	Animals and other organisms
[x]	[ ]	Human research participants
[x]	[ ]	Clinical data

Methods

n/a	Involved in the study	
[x]	[ ]	ChIP-seq
[ ]	[x]	Flow cytometry
[x]	[ ]	MRI-based neuroimaging

Antibodies

Antibodies used	Antibodies used in vivo:<br>PGT121, VRC07-523, and VRC07-523LS IgG1 antibodies were produced in Expi293F cells and purified using a Protein A column at the Vaccine Research Center, NIAID, NIH. Anti-CD8alpha depleting antibody (mouse/rhesus CDR-grafted IgG1, clone M-T807R1) was sourced from NHP Reagent Resource, NIH.<br><br>Antibody reagents for flow cytometry:<br>CD95 FITC (BD, clone DX2)<br>CD28 PE (BD, clone 28.2)<br>CD4 APC (Miltenyi, clone M-T466)<br>CD8 PB (Dako, DK25)<br>CD3 A700 (BD, clone SP34-2)<br>CD45 PE-Cy7 (BD, clone D058-1283)<br>CD20 PerCP-Cy5.5 (BD, Clone L27)
Validation	The specificity and identity of PGT121 and VRC07-523 were confirmed by discriminatory antigen binding assays in vitro. Antibodies used for flow cytometry were validated by the manufacturer and titrated on rhesus macaque blood in-house at the Vaccine & Gene Therapy Institute.

Eukaryotic cell lines

Policy information about cell lines

Cell line source(s)	TZMbl: NIH AIDS Reagent Program, catalog no. 8129. Expi293F: ThermoFisher Scientific, Inc. 293T: European Collection of Authenticated Cell Cultures.
Authentication	293T cells are sourced from the European Collection of Authenticated Cell Cultures. None of the other cell lines were authenticated.
Mycoplasma contamination	Cell lines were not tested for mycoplasma contamination.
Commonly misidentified lines<br>(See ICLAC register)	No commonly misidentified cell lines were used in this study.

Animals and other organisms

Policy information about studies involving animals; ARRIVE guidelines recommended for reporting animal research

Laboratory animals	Male and female Indian origin rhesus macaques (Macaca mulatta) were assigned randomly to study groups after confirmation of acceptable MHC allele status. Animals were aged between 3 and 4 weeks at the start of the study, and were sacrificed before 1 year of age.
Wild animals	not applicable
Field-collected samples	not applicable
Ethics oversight	The Oregon Health & Science University West Campus Institutional Animal Care and Use Committee (IACUC) approved all macaque studies.

Note that full information on the approval of the study protocol must also be provided in the manuscript.

Flow Cytometry

Plots

Confirm that:

- [x] The axis labels state the marker and fluorochrome used (e.g. CD4-FITC).
- [x] The axis scales are clearly visible. Include numbers along axes only for bottom left plot of group (a 'group' is an analysis of identical markers).
- [x] All plots are contour plots with outliers or pseudocolor plots.
- [x] A numerical value for number of cells or percentage (with statistics) is provided.

Methodology

Sample preparation	For each sample, 100 µl of whole blood was transferred from an EDTA blood collection tube into a cluster tube and washed twice with 1 ml PBS, aspirating and vortexing between washes. Surface stain antibodies and live/dead dye were then added, and samples were vortexed and incubated for 30 min at RT in the dark. Red blood cells were lysed with 1 ml of FACSLyse (1X) for 8

min, followed by 3 washes in FACS buffer. Samples were fixed in 100 ul of 2% paraformaldehyde.

Instrument	Becton-Dickinson LSR II
Software	Flowjo V9.9.5
Cell population abundance	The study used flow cytometry to measure CD4+ and CD8+ T cells in peripheral blood, which are among the most abundant cell types in peripheral blood (typically >1000 cells/µl blood for CD4+ and >500 cells/µl for CD8+).
Gating strategy	Gating strategy is shown in Supplementary Figure 8. Lymphocytes were gated by FSC/SSC, followed by singlets (FSC-A/FSC-H). Dead cells were excluded based on staining with an amine-reactive viability dye. In a SSC vs. CD45 plot, CD45+ cells were gated for further analysis. In a SSC vs. CD3 plot, T cells were defined as CD3+. This population was further refined in a CD4 vs. CD8 plot to quantify percentages of CD4+ and CD8+ single-positive cells. Boundaries between positive and negative were defined based on obvious binary separation of cell populations.

☒ Tick this box to confirm that a figure exemplifying the gating strategy is provided in the Supplementary Information.
